# Strategies of Buenos Aires Waiters to Enhance Memory Capacity in a Real-Life Setting

**DOI:** 10.3233/BEN-2008-0214

**Published:** 2009-07-29

**Authors:** Tristan A. Bekinschtein, Julian Cardozo, Facundo F. Manes

**Affiliations:** ^1^Institute of Cognitive Neurology (INECO)Buenos AiresArgentina; ^2^MRC Cognition and Brain Sciences UnitCambridgeUK; ^3^Institute of Neurosciences - Favaloro UniversityBuenos AiresArgentina

**Keywords:** Memory, real-life, expertise

## Abstract

Human learning and memory evaluation in real-life situations remains difficult due to uncontrolled variables. Buenos Aires waiters, who memorize all the orders without written support, were evaluated in situ. Waiters received either eight different orders and customers remained seated in their original locations (OL), or changed locations (CL). Match between orders, subjects and location was decreased only in CL. Waiters’ feature/location strategy links client with position at the table and beverage later. The hypothesis we raise is that memory-schemas link working memory to long-term memory networks through rapid encoding, making the information resistant to interference and enabling its fast retrieval if necessary cues are present.

## 1. Introduction

One of the most important adaptive changes in human behaviour is the learning of expertise, commonly related to the workplace. Examples include world-class chess players who have exceptional strategies for searching alternative moves from standard patterns in memory [7] or physics experts who are capable of retrieving relevant theoretical principles as a first step in solving problems. Expertise is also found in other disciplines such as writers, truck drivers and dancers [12]. The theoretical common denominator in the acquisition of various skills appears to be the use of pattern-based retrieval techniques as the backbone of the strategy. Knowledge could first be acquired and then organized into actions that, with practice, could be rapidly accessed [1,13].

Superior memory (and other cognitive domains) capacity may not only be found in "gifted" individuals but in the common population with a specific metier [12]. Adaptability of human behaviours to specific cognitive environments is attained by the development of expertise on certain key tasks, maximizing the performance in a highly constrained activity. After several hundred hours of training in a working memory task college students increased their capacity for digits from 7 to 79 [8].

In a recent study ten world-class memory experts were compared to ten control subjects matched by intelligence and spatial ability [16] showing better performance in working memory and verbal memory but no differences for visual memory. Functional imaging used during "learn order" and "detect change" revealed increased activity in memory experts in a network comprising the retrosplenial cortex, medial parietal cortex and right posterior hippocampus across tasks. The hypothesis is that this network reflects the general strategy of mental walk technique or "method of the loci" (reported after the study by nine of the memory experts), since the areas found have been commonly related to spatial memory and navigation [17]. The "method of the loci" involves the use of routes to visualize to-be-remembered items during salient points in an imaginary walk [5].

Experts who do not receive a formal education may use different strategies to achieve their high level performance. Waiters and waitresses capacity to memorize drink or dinner orders without written support rep-

ISSN 0953-4180/08/$17.00 © 2008 – IOS Press and the authors. All rights reserved

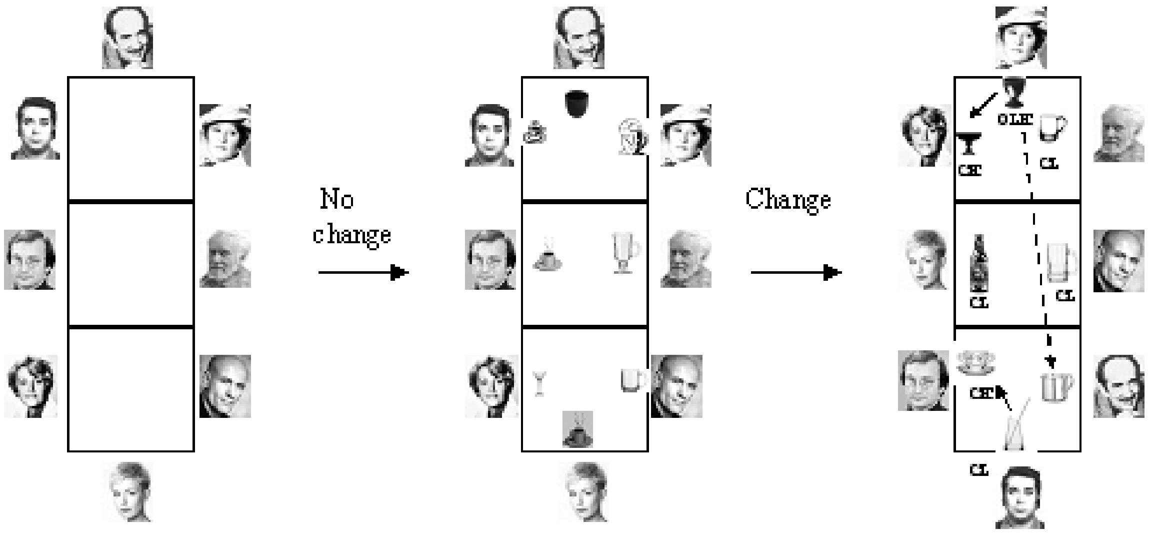

Fig. 1. Diagram (an example) of the experiment performed in the Cafes of Buenos Aires. First the customers order, the subject comes back and delivers it without errors, in the second round the customers change seats and the errors appeared.

resent a useful model to study ecological (unguided) cognitive strategies to attain expertise in everyday activities. Interviews after experimental sessions in laboratory analogues to the restaurant or bar setting, suggested that these experts encoded drink orders by establishing links between the order, person and location at the table [4,10]. They also claim that during a second call the waiters retrieved orders by using the seated location of the customers as cues to help them remember customer's orders, when the cues linked to the customers in turn might provide access to their drink orders.

Typical Buenos Aires senior waiters memorize all the orders from respective clients and take the orders without written support of as many as ten persons per table. They also deliver the order to each and every one of the customers who ordered it without asking or checking. After several years of practice these subjects become experts encompassing a unique model to study learning and memory in an ecological situation [4], a phenomenon that we called "the Tortoni effect" in honour of Cafe Tortoni, one of the most traditional bars in Buenos Aires City.

Our aim involved the study of waiters' strategy to encode, maintain and retrieve a large number of orders. We designed a controlled, single-blinded (only professional waiters were not aware) experiment to be performed in real-life settings. Eight "customers" asked for a pre-established beverage and either remained in their seats (first condition) or changed to a predefined location (second condition). Waiters' and non-experts' match performance between drink and person were calculated for both conditions, and errors classified.

## 2. Methods

It has been claimed that it takes approximately 10 years of intensive study and practice to become a world expert [6], reason why we followed this criterion to select the waiters for our study. We performed the experiment with 9 (aged 38 to 55 male) waiters who had between 9 and 17 years of experience. In a second experiment 8 non-expert male volunteers (35–55 y.o.) were selected to perform the waiters' task in an ecological laboratory setting.

The researchers (TB or JC), who acted as table coordinators, and seven participants, randomly seated around the table in each bar and instructed to be ready to make different -previously established- orders. The orders where made in a pre-fixed random sequence. We evaluated subjects' ability to deliver the correct order to the right customer in two conditions using a cross-experimental design. First, the waiter was asked for eight different drinks and the customers remained seated in their original locations (OL) at the time of the delivery of the orders. Second condition consisted in asking for eight different drinks and changing the client's locations (CL) to new, previously established seats before the waiter arrived to deliver the orders (Fig. 1). During the experiment we also took notes of the orders and customers from the other table/s each subject (waiter or control volunteer) was serving.

In the second experiment volunteers were told they would participate in an "acting task" in the bar of the hospital and should take the role of the waiter taking orders and delivering them to the right customers. Conditions were also CL and OL. A second table with two customers was added in this task. The "waiter" carried out the orders from this table with two customers after requesting orders from the table with eight customers (real-life interference).

OL performance was defined as correct match between person and beverage in the original location condition. CL performance (correct Person) was defined as a correct match, the waiter gives the order to the right (correct) person after the change. DP, the difference in performance calculated as OL-CL. OLE, Location Error: registers when the waiter failed to identify the features of the customer and assigned the item to the original location (pre- change). CE, Close error: the waiter leaves the item one position away to the original location. A: absent data, the subjects abandoned the task. The "Other tables" column is expressed as customers in table one + customers in table two + . . . ). All results are expressed as number of correct matches in the table and mean number with standard deviation in between parenthesis in the text. A nonparametric (Mann-Whitney U) test for small samples was used for comparison of DP between groups.

## 3. Results

The results showed (Table 1) that in the control condition –OL- all waiters except one (waiter A) had a perfect match between objects and subject (and location); waiter A made two mistakes since he delivered the right beverage to six of the eight customers. The group score for OL was 7.8 (0.67). In the change condition –CL- all waiters made mistakes except one (waiter I). The group score for CL was 3.1 (1.5) ranging from 1 to 6 for correct matches between beverage and customer. The number of original location errors (OLE), when the waiter leaves the beverage at the location where the customer originally made the order (pre-location change), indicates when location strategy is used, we found 0 to 3 errors for this measure. There were no obvious relationship between CLs and OLEs or CLs and ECs in the group, these two results were not associated, when waiters were unsure of the match between person and beverage they did not left the beverage in the original location (OLE) or next to it (CE). The variability in the number of tables and customers served by the waiters while serving the experiment's table acted as interference and was an uncontrolled source of variance. We found no clear association between the interference and the error measures.

Table 1
Waiters and normal volunteers' performances during no change and change of seats conditions

| | OL | CL | DP | OLE | CE | Other tables |
|---|---|---|---|---|---|---|
| *Waiters* | | | | | | |
| WA | 6 | 3 | 3 | 0 | 4 | 2 + 3 |
| WB | 8 | 1 | 7 | 1 | 1 | 2 |
| WC | 8 | 2 | 6 | 0 | 2 | 4 |
| WD | 8 | 2 | 6 | 2 | 3 | 2 + 8 |
| WE | 8 | 2 | 6 | 3 | 1 | 1 + 2 + 6 |
| WF | 8 | 4 | 4 | 1 | 2 | 3 + 2 |
| WG | 8 | 4 | 4 | 1 | 0 | 2 |
| WH | 8 | 4 | 4 | 0 | 3 | 2 + 2 |
| WI | 8 | 6 | 2 | 0 | 0 | 3 + 1 |
| *Volunteers* | | | | | | |
| VA | 6 | 3 | 3 | 1 | 1 | 2 |
| VB | 5 | 3 | 2 | 2 | 1 | 2 |
| VC | 3 | | | A | | 2 |
| VD | 4 | 2 | 2 | 2 | 2 | 2 |
| VE | 8 | 3 | 5 | 0 | 1 | 2 |
| VF | 4 | | | A | | 2 |
| VG | 4 | 2 | 2 | 2 | 2 | 2 |
| VH | 3 | | | A | | 2 |

OL: performance when all costumers stayed at their original locations. CL (correct Person): the waiter gave the order to the right (correct) person after the change. DP: The difference in performance calculated as OL-CL. OLE, Location Error: registers when the waiter failed to identify the features of the customer and assign the item to the original location (pre- change). CE, Close error: the waiter leaves the item one position away to the original location. A: absent data, the subjects abandoned the task. The "Other tables" column is expressed as customers in table one + customers in table two + . . . ).

The results in the experiment are in agreement with the verbal reports given by the waiters after the experiment. All waiters (except WI) reported the use of both location (place) where the order was given and feature (face and outfit), to deliver the order to the correct customer. WI reported the use of faces and clothes to give the beverage to the correct person since his earlier experience had been mostly in cocktail parties for 10 years, where people tend to change their position in the room; only in the last three years he had been working in the restaurant.

Volunteers' performance was lower than their expert counterparts in the waiter's task. Only one subject delivered the eight orders correctly to the right customers, another made only one mistake and the others made three mistakes or more (Table 1). Results in CL were similar to the experts'. Three subjects left all the beverages on the table and decided to leave the task unfinished. In the post-experiment interview these three volunteers stated that OL was difficult but CL was (far) too difficult and confusing to be completed. The other five subjects registered a variety of errors that didn't differ from the expertise group.

To measure the effect produced by the change of seats in our two groups of subjects we calculated the Difference in Performance (DP) by subtracting OL to CL. DP was 4.6 (1.6) for waiters and 3 (1.1) for novice subjects. A nonparametric (Mann-Whitney U) test for small samples showed a significant difference ($p = 0.043$) between groups for DP.

## 4. Discussion

Buenos Aires traditional waiters demonstrated an expert memory for drink orders in an ecological controlled experiment, as compared to a group of control novice subjects, suggesting a bound strategy (both spatial and feature based) at the core of the expertise.

Expert individuals have a rich background of relevant experience and therefore can organize new material into meaningful and orderly patterns using "schemas". This concept refers to pre-existing knowledge structures into which newly acquired information can be incorporated [3]. Following this stream of thought waiters can have superior memory for drink and dinner orders [4, 10] and the expertise to link these to the client's face and location would be the schema. A second memory feature these experts might use, when possible, is pattern-based chunking strategies (i.e. soft drinks for these two customers and alcohol for these other two), since some errors made were beverage inversions (e.g. cafe latte instead of black coffee, and vice versa). Collected verbal reports from our skilled waiters support this encoding method.

The neural correlates of expanded memory capacities have been scarcely studied. Taxi drivers, a model of expert navigation, showed larger posterior hippocampi relative to those of control subjects and hippocampal volume also correlated with expertise [18]. A famous expert in calculation – Mr. Gamm- recruited a system of brain areas implicated in episodic memory (right medial frontal and parahippocampal gyri) while performing a calculation task whereas control subjects did not [19]. The study suggests that experts may be able to use the unlimited storage capacity of long-term memory to maintain task-relevant information for complex calculation, whereas the rest of us rely on the very limited span of working memory. However, brain activity results in world-class memory experts showed a network related to navigation but not to episodic memory.

Classic approaches for the study of expertise comprise the detailed characterization of outstanding individuals and the comparison of skills between groups of experts and novices. Famous cases of mnemonists in the history of neuroscience include Mr. Shereshevskii, who was able to "remember any information presented" [15]. He used three strategies: first he generated rich visual images to represent the information; second he used the loci method; and third, he created a story with the information using appropriate images to facilitate retrieval. An outstanding characteristic of S was that he unconsciously used his synesthesia to encode information. Mr. Rajan, who can recall ten thousand digits of $\pi$, encodes the digits in chunks and attaches cues for retrieval of the chunk; but his memory span for symbols and digits do not differ from normal volunteers [11]. Single case studies suggest that experts use various techniques to remember [14].

Ericsson's theory of skilled memory proposed three general principles: the use of pre-existing knowledge to store information, cued attachment to encoded information to facilitate retrieval, and the reduction in time of encoding with practice [9]. The famous experts displayed different memorizing strategies but all used cues to enhance retrieval and showed a significant reduction in the time spent to study. But Rajan and S did not use pre-existing knowledge as a key feature of their strategy and thus the theory it is not fully supported by the single case studies. Lastly, another case study, JC, a waiter who could recall as many as 20 dinner orders [10], categorized the food (meat or starch) and linked it to the location in the table. He also used acronyms and words to encode salad dressing and visualized cooking temperature for each customer's meat and linked it to the position on the table. Contrary to the waiters in our study, JC trained himself and developed strategies to achieve his expertise level; Buenos Aires waiters reported systematically that they have not thought of any particular strategy and that their great ability comes only with time and practice. They have developed their expertise spontaneously as suggested by the debriefing of the interviews. Although this extended-in-time working memory (lasting several minutes) has been previously reported [9,10], our study is the first to address directly the effect of interference and change of context (spatial rearrangement of customers) when the expertise is taking place. The results suggest that all waiters except WI use a bound strategy (spatial and feature based) that allows them to form associations between the beverage, the place, and the person.

The cognitive processes performed by the waiters seem to exceed the standard definition of working memory referred to as "the temporary storage of information that is being processed in any of a range of cognitive tasks" [2] since the retrieval occurs several minutes after the order is taken. Remarkably our subjects took orders from other tables and despite this interference; they gave the beverages to the right customer (in the control condition). In the normal volunteer group the performance in the OL condition was lower than the experts probably due to the lack of expertise but also because five of them reported that the table for two (interference table) increased the difficulty of the task. Interestingly waiters did not pay attention to any customer after taking a table's order as if they were protecting the memory formation in the path from the table to the bartender or kitchen.

One of the proposed hypotheses to account for working memory long maintenance and resistance to interference is the rapid consolidation of new information as a result of a pre-existing schema [20]. It has been recently shown that mice can create schemas by learning to associate six flavours with six places in the course of six weeks training and then acquire new flavour-place associations in a single trial and remember them for two weeks [21]. The hypothesis we raise is that memory schemas link working memory to long-term memory through rapid encoding, making the information resistant to interference and enabling its fast retrieval.

This study goes beyond typical instruction and lab context effects because it lacks all lab constrains. It is therefore a true real-life experiment and has allowed us to evaluate memory capacity and strategy in a natural setting. Unfortunately there are several factors that cannot be controlled such as the amount of other tables the waiters take orders during the experiment and the time between the order and the delivery. Although greater efforts should be done to evaluate human cognition in ecological situations, this experimental design only allows for limited conclusions.

Several studies have suggested that spatial strategy confers an advantage in the encoding and retrieval of specific material [12]. Most of these results are based on questionnaires and interviews; our results provide empirical evidence that memory expert waiters use not only spatial cues but also specific features of the client to accomplish their everyday tasks.

In summary, we suggest that the waiters used a bound (feature- location) strategy and that this expertise might be learnt using memory schemas allowing experts to easily retrieve information if the necessary cues are present. Perhaps this strategy is commonly used by people in everyday life routine situations.

## Acknowledgment

We thank Mitul Metha and Milena Winograd for useful comments and for being table coordinator in the pilot study of the project. We also thank the Neurocenas group and friends for their participation as customers. We specially thank the waiters.

## Expertise Questionnaire and instructions

1. Sir, thank you for your patience, in fact we were running a memory experiment while you were serving us. (Allow reply).
2. We are interested in the increased memory capacity of experienced waiters which allows them to recall the order of every person in the table and deliver it correctly later. (Allow reply).
3. How much experience do you have as a waiter?
4. How many tables were you serving besides ours?
5. Do you have any method or strategy to do your job?
6. Have you ever been trained as a waiter?
